# Insights into Tardigrade Damage-Suppression Protein, Dsup

**DOI:** 10.3390/biom16030455

**Published:** 2026-03-18

**Authors:** Tyler J. Woodward, M. Todd Washington

**Affiliations:** Department of Biochemistry and Molecular Biology, College of Medicine, University of Iowa, Iowa City, IA 52242, USA; tjwoodward@uiowa.edu

**Keywords:** DNA damage, genome stability, intrinsically disordered protein, protein-DNA interactions

## Abstract

Tardigrades are microscopic invertebrates capable of surviving extreme environmental conditions through unique molecular adaptations. Among the proteins implicated in their remarkable resilience is a novel protein known as damage suppressor (Dsup), a key factor in protecting cellular DNA from elevated levels of radiation. Since its discovery, numerous studies have explored the biochemical, structural, and functional properties of Dsup. In this review, we summarize the current knowledge surrounding these properties and describe several proposed mechanisms by which Dsup may confer protection. For each proposed mechanism, we outline the foundational model, present supporting evidence, and highlight critical gaps in our understanding. Taken together, we believe that Dsup likely employs multiple complementary mechanisms to protect DNA. Finally, we discuss emerging applications of Dsup and Dsup-inspired technologies for human health. Overall, this review synthesizes our current understanding and provides a framework to guide future investigations into this remarkable protein.

## 1. Introduction

DNA damage is caused by a variety of physical and chemical agents [1,2,3]. For instance, ionizing radiation is a physical agent that causes numerous types of base damage as well as single- and double-stranded breaks [4,5,6]. Another example is ultraviolet (UV) radiation, which causes cyclobutane pyrimidine dimers and pyrimidine-pyrimidone photoproducts [7,8,9].

Chemical agents also cause DNA damage, with reactive oxygen species (ROS) as a prominent example that cause oxidative base lesions such as 8-oxoguanines [9,10]. Polycyclic aromatic hydrocarbons and aromatic amines cause specific DNA lesions such as benzo[*a*]pyrene *anti* diol-epoxide (BPDE)-derived adducts and acetyl-aminofluorene (AAF)-derived adducts, respectively [11,12]. If left unrepaired, such DNA damage can interfere with DNA replication leading to mutations and genomic rearrangements that drive carcinogenesis, aging, and a wide variety of other diseases [2,6,8,10,11,12,13]. Consequently, there has been extensive effort to understand how organisms detect, repair, and otherwise tolerate DNA damage.

Some organisms are more tolerant of DNA damaging agents than others [14]. Among the organisms most tolerant to radiation are tardigrades—microscopic invertebrates renowned for their ability to survive extreme environmental conditions [15,16,17,18,19]. Tardigrades can tolerate extreme temperatures ranging from −273 °C to over 100 °C, extreme pressures as high as 1200 atm, desiccation, and immersion in organic solvents [15,16,17,18,19]. Likewise, tardigrades can tolerate extreme levels of radiation as high as 5000 Gy [15,20,21]. For the sake of comparison, whole-body radiation doses of 5 Gy are often lethal to humans without medical care [22]. These properties make tardigrades powerful model organisms for studying cellular resilience. Their remarkable extremotolerance is thought to be the result of a diverse repertoire of molecular adaptations that maintain protein and DNA integrity under such extreme conditions [15,16,17,18,19,20,21].

A key breakthrough in our understanding of the extremotolerance of tardigrades occurred in 2016 when researchers from the University of Tokyo identified several genes unique to *Ramazzottius varieornatus*—a tardigrade species noted for its exceptional resistance to radiation [23]. One of these genes encoded a protein comprising 445 amino acid residues with little to no sequence homology to any known protein. When this gene was constitutively expressed in human embryonic kidney 293T (HEK293T) cells, it reduced the amount of radiation-induced DNA damage as assessed by single-cell gel electrophoresis assays (i.e., comet assays). Based on these finding, the researchers named this protein damage suppressor, or Dsup [23]. Given its lack of sequence homology to other known proteins and its ability to confer radiotolerance to mammalian cells, Dsup has garnered considerable attention in recent years.

In this article, we discuss the key findings that have contributed to our current understanding of the cellular, functional, structural and biochemical properties of Dsup. We also describe several proposed models that explain how Dsup may confer genomic protection from DNA damaging agents. These include mechanisms that help prevent DNA damage directly such as physically shielding, altering the hydration state, and inducing a conformational change in the DNA to make it less susceptible to damage. These also include mechanisms that help indirectly such as assisting DNA repair proteins locate DNA damage and acting as a molecular scaffold to increases the efficacy of DNA repair proteins. We will present the supporting evidence for each model and highlight critical gaps in our understanding. Finally, we discuss emerging applications of Dsup and Dsup-inspired technologies for human health. Overall, this review synthesizes our current understanding of and provides a framework to guide future investigations of this remarkable protein.

## 2. Cellular, Functional, Structural, and Biochemical Studies of Dsup

Since the discovery of Dsup ten years ago, multiple papers studying the heterologous expression and characterizing the structural and biochemical properties of Dsup have been published [23,24,25,26,27,28,29,30,31,32,33,34,35,36,37,38,39,40]. In this section, we will discuss a series of studies showing that Dsup retains its ability to protect cells from radiation-induced DNA damage in a wide range of eukaryotes. We will also discuss what is currently known about the structure and DNA-binding activity of Dsup.

### 2.1. Cellular and Functional Studies of Dsup

Several studies have shown that Dsup elicits similar protection from radiation-induced DNA damage when it is expressed in various heterologous eukaryotic systems [23,24,25,26,27,28,29,30,31,32,33,34]. These include systems such as human, yeast, roundworm nematodes, fruit flies, rice, and tobacco [23,24,25,26,27,28,29,30,31,32,33,34]. Interestingly, while Dsup provides radioprotection in HEK293T cells, its expression in human primary neuronal cells has been reported to induce DNA damage, particularly double-strand breaks, even in the absence of stress [26]. The cause of this differential effect remains unclear, highlighting the need for more mechanistically focused cellular studies.

Beyond its role in protecting DNA from the direct effects of ionizing radiation, Dsup also provides protection against the indirect effects of such exposure and from non-ionizing radiation [34]. For example, ROS (common byproducts of ionizing radiation) and UV-C (a type of non-ionizing radiation) can both damage DNA. It has been shown that Dsup protects DNA from ROS in both yeast and human cells [25,27,29,34]. Furthermore, it has been demonstrated that Dsup protect DNA from UV-C radiation in human cells [25]. Interestingly, Dsup provides little to no protection from ultraviolet radiation in yeast [29]. These results demonstrate Dsup provides protection to DNA against various insults including the direct and indirect effects of ionizing radiation, as well as non-ionizing radiation. Again, these results reinforce the need for more mechanistically focused studies across multiple eukaryotic systems.

In addition, several research groups have focused on the potential ability of Dsup to influence transcription. In fact, Dsup was initially thought to be a non-specific global repressor of transcription [28]. However, recent studies (using RT-qPCR) have demonstrated that heterologous expression of Dsup selectively alters gene transcription by upregulating the expression of DNA repair and cell cycle checkpoint proteins such as PARP1, BRCA1, BRCA2, RAD50, RAD17, and ATM [25,27,28,30]. Further studies have shown that the effects of heterologous expression of Dsup can differ with respect to environmental conditions. For example, upon exposure to ROS, Dsup expression is correlated with transcriptional downregulation of genes encoding DNA repair proteins such as XRCC6, ERCC6, and RAD1. By contrast, upon exposure to ultraviolet radiation, Dsup expression is correlated with transcriptional upregulation of genes encoding some DNA damage response proteins (such as XRCC6, ERCC6, ATR, and BRCA1) and transcriptional downregulation of genes encoding other DNA damage response proteins (such as BRCA2 and ERCC1) when examined via RT-qPCR [25].

Despite the reports of selective transcriptional regulation by the heterologous expression of Dsup, significant gaps in our knowledge remain. For instance, it is unclear how Dsup expression selectively alters the transcription of genes in response to diverse types of DNA damage. Furthermore, it is unclear how the responses to Dsup expression vary among different eukaryotic species. Finally, it is unknown to what extent any of these transcriptional changes can account for the magnitude of protection from DNA damage observed when Dsup is heterologously expressed in these systems. It seems highly unlikely that such changes in gene expression can fully account for the extreme radiotolerance conferred by Dsup. Likely any increases in radiotolerance due to transcriptional changes are in addition to increases due to the various possible mechanisms discussed below.

### 2.2. Structural Studies of Dsup

As mentioned above, Dsup comprises 445 amino acid residues with little to no sequence homology to any known protein, aside from a short high mobility group nucleosome binding domain-like (HMGN-like) motif spanning residues 363 to 369. While there is a lack of homology to any known proteins, a Dsup ortholog was identified in *Hypsibius exemplaris*, a tardigrade subspecies [34]. While *H. exemplaris* Dsup (He Dsup) has a HMGN-like motif, it only has ~26% similarity in amino acid sequence to *R. varieornatus* Dsup (Rv Dsup) and is shorter than Rv Dsup (328 residues compared to 445 residues, respectively). Although He Dsup shares similar characteristics of Rv Dsup, very few studies of He Dsup have been conducted. For this reason, we will focus exclusively on Rv Dsup in the article.

Dsup has unusual primary sequence. It has almost no aromatic residues—there are two phenylalanine residues, one tyrosine residue, and no tryptophan residues. In addition, Dsup contains no cysteine residues and thus cannot form intramolecular or intermolecular disulfide bonds. Dsup is also highly positively charged with a net charges of +23 at neutral pH. Lastly, Dsup has been shown to bind free DNA, free RNA, and histone proteins [23,28,33,40]. It is likely that the positive charges of Dsup are involved in electrostatic interactions with the DNA [23,36,37].

**Figure 1:**
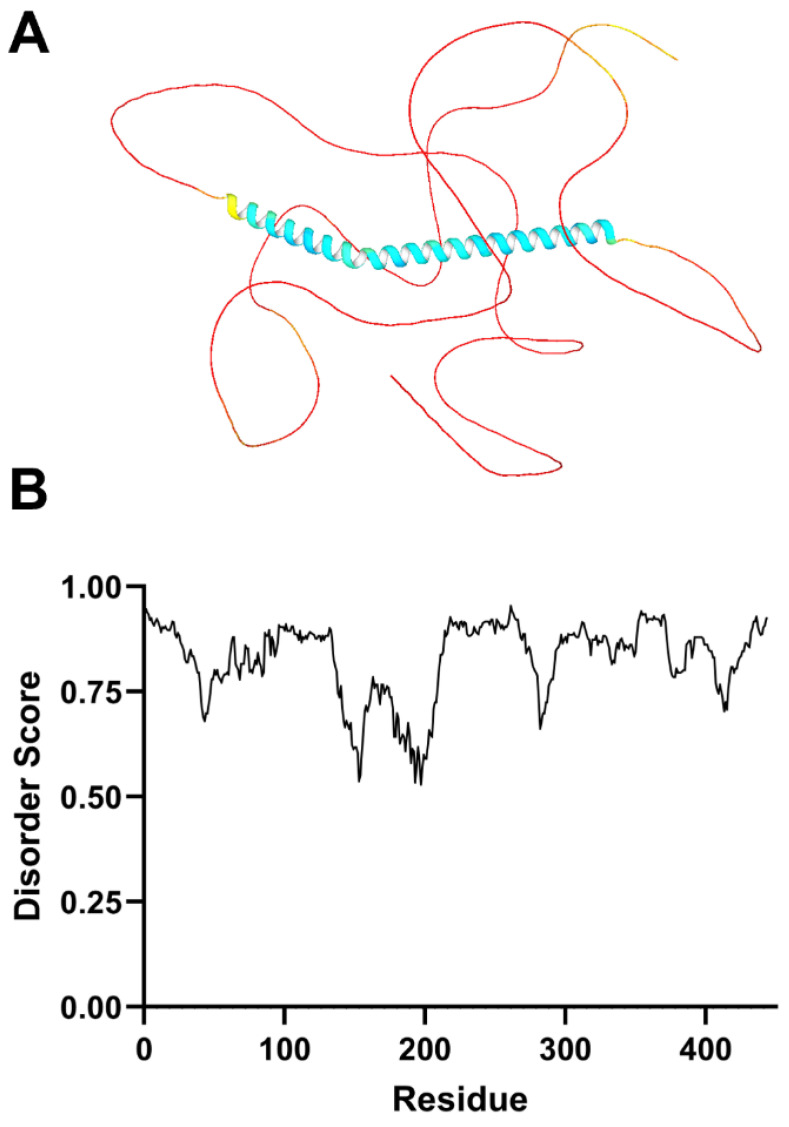
Structural Predictions of Dsup. (**A**) Cartoon representation of the AlphaFold3-predicted structure of Dsup [43]. Regions in red indicate low-confidence predictions (pLDDT < 50), while regions in cyan indicate moderate-to-high confidence (70 < pLDDT < 90). (**B**) Intrinsic disorder profile of Dsup generated from its primary amino acid sequence using IUPred2A [41,42]. Values above 0.5 are predicted to be disordered.

Dsup is believed to be almost entirely disordered [36,37,38,39,40]. Over 60% of its primary sequence consists of serine, alanine, glycine, and lysine residues—amino acids known to be enriched in intrinsically disordered proteins. Consistent with this, computational analysis using IUPred2A predicts Dsup to be almost entirely disordered (Figure 1A) [41,42]. Similarly, AlphaFold 3 predicts Dsup is entirely disordered except for residues 144 to 208 which are predicted to form an α-helix (Figure 1B) [43]. Aside from this putative helix, Dsup appears to be devoid of secondary and tertiary structures when analyzed by predictive computational algorithms.

To experimentally determine if Dsup is indeed both intrinsically disordered and conformationally flexible, two groups, which included our group, studied Dsup using small-angle X-ray scattering (SAXS) [38,39]. While both groups obtained similar experimental SAXS data and conclusions, they analyzed the data in very different ways. The first group used Kratky analysis to show that Dsup is predominantly disordered [38]. They also carried out 3D modeling of Dsup using DAMMIF and DAMAVER to show that Dsup is monomeric and extremely extended compared to what would be expected from a more folded protein. To compliment the scattering analysis, this group used circular dichroism to show that over 60% of Dsup is a random coil [38].

Our group used a full-ensemble method to validate a course-grained Brownian dynamics simulation of Dsup with the experimental SAXS data [39]. Utilizing a full-ensemble method, we found excellent agreement between the Rg and Dmax values from the analysis of the simulations and SAXS data. Furthermore, the simulations demonstrated Dsup is entirely disordered and samples a vast region of conformational space. To compliment the scattering analysis, we also used mass photometry to show that Dsup exists in solution primarily as a monomer. Moreover, we also used infrared spectroscopy to show that over 50% of Dsup is disordered [39]. Taken together, these two biophysical studies experimentally demonstrate that Dsup is predominantly intrinsically disordered, mostly monomeric, and conformationally flexible in solution.

Dsup has an HMGN-like motif spanning residues 363 to 369. In the HMGN2 protein, the consensus motif is important for binding to nucleosomes and opening the chromatin structure to allow better access to transcription factors and DNA repair proteins [44,45,46]. In HMGN2, two arginine residues interact with the acidic patch of the nucleosome. The first arginine residue (arginine-23) interacts with glutamate-56 on histone H2A and with glutamate-113 on histone H2B, and the second (arginine-27) interacts with glutamate-61, aspartate-90, and glutamate-92 of H2A [40]. These interactions with the acidic patch effectively anchor the HMGN2 protein to the nucleosome.

**Figure 2:**
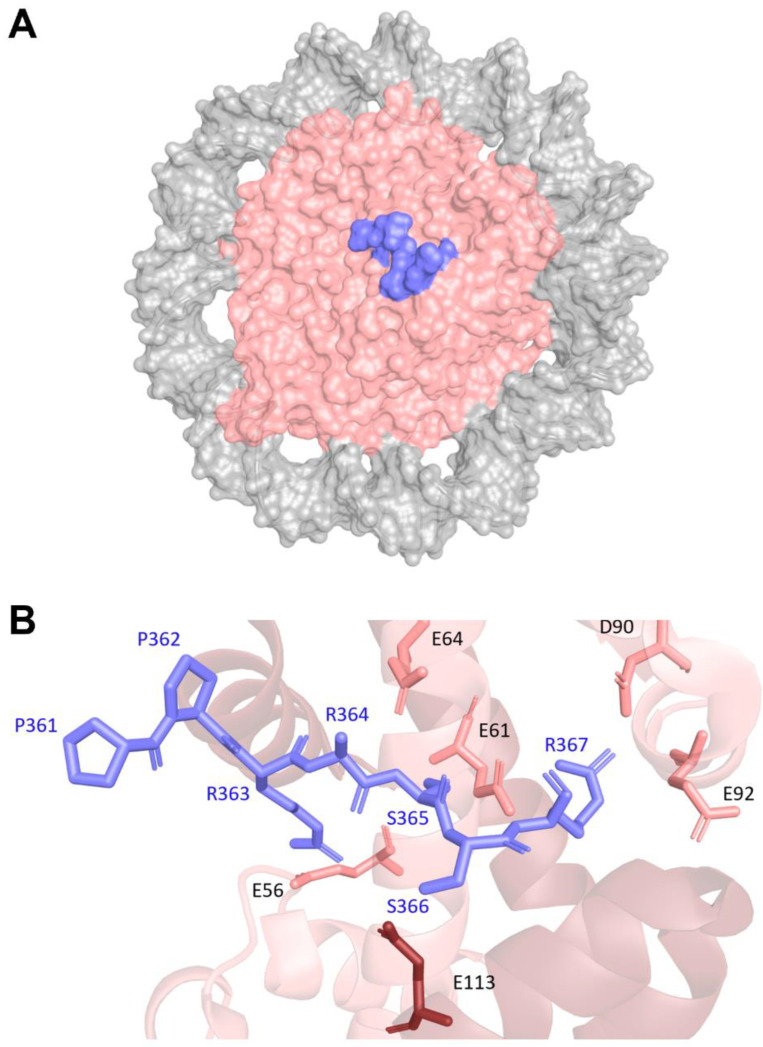
Nucleosome-Bound Dsup Structure. (**A**) Cryo-electron microscopy (cryo-EM) structure of Dsup bound to a nucleosome, solved at 2.7 Å resolution (Protein Data Bank, PDB: 9D3K) [40]. Dsup (slate blue) binds the acidic patch of the histone core (salmon), while the nucleosomal DNA (gray) adopts a closed conformation. (**B**) Detailed view of the Dsup–nucleosome interface. Dsup (slate blue) interacts with histones H2A (light salmon) and H2B (dark salmon). Key interacting residues from both Dsup and the histone proteins are indicated.

Recently, the structure of Dsup bound to a nucleosome core particle was determined by cryo-electron microscopy [40]. Interestingly, while the entire Dsup protein was used to determine this structure, electron density for only residues 361 to 369 is present in the structure (again suggesting extreme flexibility and lack of structure in Dsup). This region corresponds to the HMGN-like motif of Dsup. As expected, this region was bound to the acidic patch on the nucleosome (Figure 2A). The mode of interaction between the HMGN-like motif of Dsup and the acidic patch is similar to the interaction between HMGN2 and the acidic patch [40]. The first arginine residue of the Dsup motif (arginine-363) interacts with glutamate-56 on histone H2A and with glutamate-113 on histone H2B, and the second (arginine-367) interacts with glutamate-61, aspartate-90, and glutamate-92 of H2A (Figure 2B). No other well-defined structural regions of Dsup are observed bound to any other positions on the histones or the DNA of the nucleosome, demonstrating that the majority of Dsup likely remains unstructured and dynamic.

### 2.3. Biochemical Studies of Dsup

Initial electrophoretic mobility shift assays showed that Dsup directly binds to DNA and RNA [23,28]. Recently, our group carried out a systematic analysis of the DNA-binding properties of Dsup [39]. Using biolayer interferometry, the K_d_ for Dsup binding various DNA ligands was determined by measuring the equilibrium response units as a function of Dsup concentration. The kinetics of binding, however, were not analyzed because unless the binding mechanism is one step, the kinetics of binding is difficult to properly interpret from biolayer interferometry. This caveat in no way effects the accuracy of the K_d_ determinations. It was shown using biolayer interferometry that Dsup binds both single-stranded DNA and double-stranded DNA with high affinity with a K_d_ value in the low nanomolar range (<10 nM). Furthermore, Dsup binds DNA molecules containing DNA damage—specifically abasic sites and 8-oxoguanines—with the same affinity that it binds non-damaged DNA. In addition, Dsup binds double-stranded DNA of differing lengths with similar high affinity (<10 nM) provided that the DNA is at least 30 base pairs long. Dsup binds shorter DNA molecules with lower affinity (>10 nM), which suggests that the minimal DNA length for optimal Dsup binding is approximately 20 to 30 base pairs [39].

A set of N-terminal and C-terminal deletion constructs of Dsup were used to determine which regions of Dsup are important for DNA binding. Both an N-terminal fragment of Dsup (residues 1 to 270) and a C-terminal fragment (residues 270 to 445) each bind DNA with 200-fold to 300-fold lower affinity than full-length Dsup [39]. These results, along with additional data, show that residues throughout the entirety of Dsup contribute toward its ability to bind DNA. This has led to the hypothesis that Dsup forms a “fuzzy” complex with DNA, which means that Dsup interacts with DNA through transient and multivalent interactions distributed across the entirety of the Dsup protein.

Finally, several biophysical approaches such as infrared spectroscopy, circular dichroism, and fluorescence spectroscopy indicate that Dsup alters the conformation of double-stranded DNA and itself upon DNA binding. As mentioned above, circular dichroism and infrared spectroscopy showed that Dsup is at least 50% to 60% disordered when not bound to DNA [38,39]. Upon DNA binding, both techniques showed that the α-helical content of Dsup increases slightly. For example, circular dichroism showed that the α-helical content increases from about 5% to 10% [38].

Perhaps more interesting from a mechanistic point of view is that fluorescence and infrared spectroscopy showed that upon Dsup binding, the DNA becomes partially unwound [39]. For instance, using an internal 2-aminopurine nucleobase (an adenine analog which is fluorescently sensitive to base pairing) in a short (40 bp) DNA substrate, researchers demonstrated that Dsup partially unwinds DNA or at least alters the chemical environment around DNA bases [39]. In infrared spectroscopy experiments using microfluidic modulation, researchers found a slight decrease in absorbance corresponding to base-pairing when Dsup bound to a short (40 base pairs) DNA substrate [39]. It should be mentioned that the observed unwinding is likely only partial and transient. Nevertheless, this has led to the hypothesis that Dsup stabilizes DNA in a underwound state that may be more resistant to forming specific lesions—such as cyclobutane pyridine dimers and pyrimidine-pyrimidone photoproducts. For instance, the [2+2] photocycloaddition reaction that creates cyclobutane pyridine dimers requires a distance of 4.2 Å or less between adjacent pyrimidines [47]. If Dsup unwinds the DNA, the distance between adjacent pyrimidine bases may be further than 4.2 Å and thus be less likely to be photoreactive. Further research is required to directly test this idea.

## 3. Possible Mechanisms of Dsup

**Figure 3:**
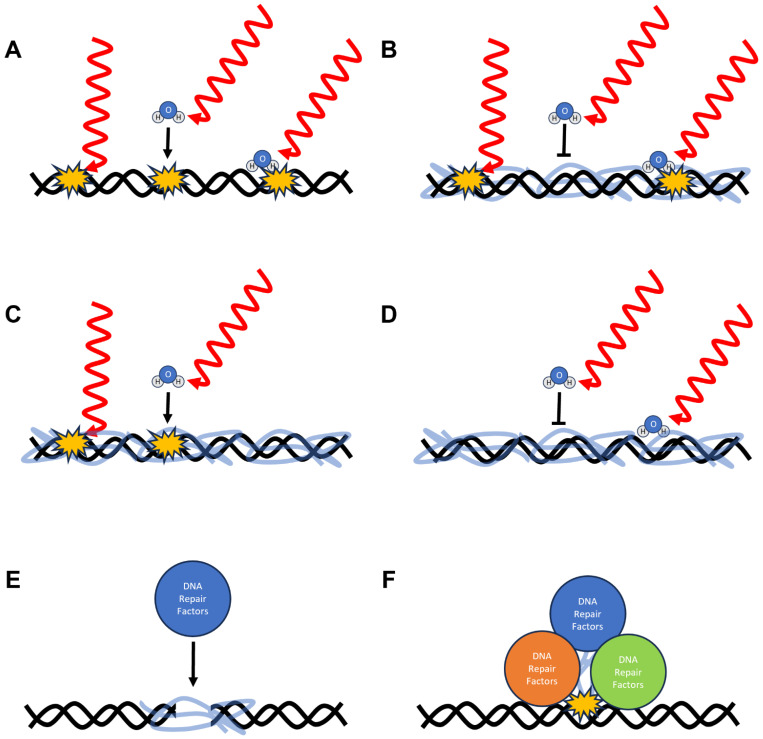
Proposed mechanisms of Dsup-mediated protection from radiation-induced DNA damage. DNA is shown in black, and water molecules are depicted as H_2_O. Radiation is represented by red wavy lines, and sites of DNA damage are indicated by yellow bursts. Dsup is represented by the clear blue ribbon. Panels (**B**–**D**) illustrate direct protective mechanisms, while panels (**E**,**F**) depict indirect mechanisms. (**A**) In the absence of Dsup, DNA damage occurs via three pathways: (1) direct ionization of the DNA, (2) generation of reactive oxygen species (ROS) from bulk water, and (3) generation of ROS from water molecules within the grooves of DNA. (**B**) Direct shielding by Dsup prevents ROS generated in bulk water from diffusing into the DNA grooves, though direct ionization and groove-generated ROS can still cause damage. (**C**) Alteration of the DNA hydration state by Dsup reduces ROS formation in the grooves but does not prevent direct ionization or ROS from bulk water. (**D**) Conformational changes in DNA induced by Dsup protect against all three types of damage by reducing DNA’s physical and chemical susceptibility. (**E**) Covalent crosslinking of Dsup to DNA indirectly facilitates repair of double-stranded breaks. (**F**) Dsup can act as a molecular scaffold, recruiting and organizing DNA repair factors at sites of preexisting damage.

Overall, there are many models/hypotheses for how Dsup confers protection to DNA (Figure 3). The various hypotheses can be divided into two broad categories: direct and indirect protection mechanisms. Direct protection mechanisms are ones in which Dsup is directly responsible for preventing DNA damage from occurring. These mechanisms include direct shielding, alterations in the hydration state, and induced conformational changes in DNA. Indirect protection mechanisms are ones in which Dsup aids or enhances other cellular mechanisms to mitigate DNA damage. These mechanisms include identifying sites of DNA damage and acting as a molecular scaffold for DNA repair factors. As mentioned above, DNA damage transcriptional control could be involved in these indirect mechanisms. Here, we will present supporting evidence and point out potential problems for each model. These hypotheses are not mutually exclusive, and the actual basis of DNA protection likely involves a combination of several of these models.

### 3.1. Direct Shielding

One of the most commonly proposed direct mechanisms of Dsup action is physical shielding of DNA. In this model (Figure 3B), Dsup binds extensively along the DNA backbone when it is bound to nucleosomes or when it is free. This effectively could result in Dsup forming a dynamic, protective coating around the genome. It is hypothesized that this coating would reduce the likelihood that the DNA would be damaged by reactive oxygen species and other DNA-damaging products created by ionizing radiation. It should be noted that the unstructured and dynamic nature of Dsup as implied by the cryo-EM study [40] does not invalidate this model. In fact, it may lead to more effective shielding. For instance, the large intrinsically disordered regions of Dsup may increase the mean diffusional path length for DNA-damaging compounds to reach the DNA, effectively “hiding” it from these extremely short-lived chemical species.

Several lines of evidence support this model. First, Dsup has been shown to bind nucleosomes and DNA with high affinity and that it does so via multivalent interactions [39,40]. Such binding activity is consistent with the formation of an extensive yet transient DNA–protein complex. Second, cellular studies suggest that Dsup is constitutively expressed and in high abundance [23], implying that it is associated with substantial portions of chromatin. Furthermore, Dsup has been demonstrated to bind to various types of DNA, including various lengths and types of DNA damage [39]. This type of non-specific DNA-binding would be required to effectively coat the diverse structures found in the genome of a cell. Finally, the direct shielding model provides a unified explanation for why Dsup expression protects a wide range of eukaryotic cells from diverse insults—including ionizing radiation, UV radiation, and oxidative stress—each of which damage DNA through distinct mechanisms [1,2,3,4,5,6,7,8,9,10,11,12,13,14,23,25,27,29,34].

Despite this evidence, significant uncertainties remain. The degree to which Dsup occludes DNA within these complexes is unknown. In addition, “fuzzy” complexes are typically transient. If Dsup forms such dynamic complexes with DNA, continuous high-level expression would likely be required to maintain protection. Constitutive high expression, however, could impose a high metabolic cost. Finally, to coat the DNA fully, Dsup would likely need to bind with high stoichiometry, likely requiring high positive cooperativity. To date, there are no studies demonstrating high stoichiometry or positive cooperation for DNA binding by Dsup. Consequently, while the direct shielding model is attractive and consistent with much of the available data, it remains to be fully fleshed out through quantitative and structural studies measuring the extent, stability, and functional consequences of Dsup–DNA complexes.

### 3.2. Alteration to the Hydration State

Another proposed direct mechanism is that Dsup alters the hydration shell of DNA, thereby dehydrating the DNA and reducing the probability of radiation-induced base lesions and strand breaks. In this model (Figure 3C), the intrinsically disordered regions and charged residues of Dsup interact with the phosphate backbone of DNA and the surrounding solvent. These interactions would reorganize the local environment of the DNA, effectively displacing much of the water in the immediate vicinity of the DNA. By doing so, Dsup could limit the formation of hydroxyl radicals near the DNA and reduce the likelihood of radiation-induced lesions.

Several lines of evidence lend support to this hypothesis. First, Dsup is a highly hydrophilic and charged protein, characteristics known to influence hydrogen bonding and electrostatic interactions in its local solvent environment [23,36,37]. Second, molecular dynamics simulations and spectroscopic analyses of other intrinsically disordered, DNA-binding proteins show that changes in local hydration can stabilize DNA and reduce susceptibility to radiation-induced damage [36,39]. In addition, tardigrades are capable of anhydrobiosis, a cellular mechanism that substantially reduces the intracellular water content [18,19]. Previous studies suggest tardigrade-unique disordered proteins are responsible for the removal of water [48,49]. It is possible Dsup is facilitating or aids this process in the nucleus. Finally, Dsup has been demonstrated to alter the conformation of DNA when bound. This change in conformation could lead to the removal of structured water in the major and minor grooves of DNA, thus reducing the likelihood of ROS formation near the DNA.

Despite this, there is little experimental evidence demonstrating that Dsup measurably alters DNA hydration or solvent organization. No high-resolution structural or biophysical studies have quantified water coordination in Dsup–DNA complexes. Moreover, altering hydration on a genome-wide scale would likely require high concentrations of Dsup, raising questions about whether such protein levels are achievable in vivo. Thus, while this model remains plausible and is consistent with the little evidence we have, this model awaits further testing and verification.

### 3.3. Induced Conformational Changes in DNA Structure

The final proposed direct mechanism is that Dsup induces structural changes in DNA that make it inherently more resistant to damage. In this model (Figure 3D), rather than passively shielding DNA, Dsup could actively modify DNA conformation—such as increasing DNA stretching, altering the widths of the major or minor grooves, or changing base-pair and base-step geometries—to reduce accessibility to or chemical reactivity with DNA-damaging agents. Through multivalent and electrostatic interactions, Dsup may stabilize nucleosome-bound DNA or promote transient DNA bending, wrapping, or partial unwinding in nucleosomal DNA or free DNA, effectively reducing the ability of the DNA to react with DNA-damaging agents.

Supporting evidence for this model comes from the nucleosome-binding properties of Dsup by its HMGN2 motif [40]. Due to the presence of this motif, Dsup associates with chromatin [40]. Because other disordered chromatin-binding proteins—such as linker histones and some chromatin architectural proteins—induce conformational changes in the DNA, Dsup, by analogy, could play a similar DNA-remodeling role. Recently, circular dichroism and infrared spectroscopy experiments indicate that Dsup alters the conformation of DNA leading to reduced base pairing that is consistent with partial DNA unwinding or underwinding [38,39].

Although this model is a likely explanation for some of the protection observed in the presence of Dsup, clear gaps in our understanding remain. For instance, there is no direct structural evidence showing that Dsup binding changes the conformation of DNA. In the cryo-EM structure of Dsup bound to a nucleosome, no clear density corresponding to regions of contact between Dsup and the DNA in the nucleosome is observed [40]. While it is clear how partial DNA unwinding or stretching would increase the separation between adjacent nucleotides leading to fewer ultraviolet-induced thymine dimers and pyrimidine-pyrimidone photoproducts, it is unclear how such changes in the DNA would decrease its susceptibility to damage by ROS. Furthermore, it is unclear the extent of the conformational change in DNA and whether the change significantly reduces the likelihood of DNA damage. Consequently, while DNA remodeling probably contributes to Dsup-mediated protection, it is unlikely that it is the sole mode of Dsup action.

### 3.4. Protein–DNA Crosslinking

The first possible, indirect mechanism for DNA protection by Dsup is through the formation of protein-DNA crosslinks. In this model (Figure 3E), Dsup forms multivalent interactions with DNA and chromatin prior to radiation exposure. When cells are exposed to radiation, Dsup can become covalently crosslinked to genomic DNA. If the network of Dsup proteins bound to the DNA are crosslinked both upstream and downstream of any resultant double-strand breaks, these covalent crosslinks could act as a “molecular stitch” holding the two broken ends of DNA together. This stitch would essentially aid in DNA repair by limiting the distance the two ends could diffuse apart from each other. Once the break is repaired by end joining, the protein–DNA crosslinks can be readily removed through other repair pathways. Importantly, this mechanism can also be facilitated without protein–DNA crosslinking, if Dsup binds substantially and with high affinity creating a non-covalent “stitch”.

Support for this model comes from prior findings that protein–DNA crosslinks can preserve genome stability in some contexts rather than contributing to instability [50,51,52,53]. In fact, several groups have shown that protein–DNA crosslinks play a protective role in preserving genome integrity when the DNA is exposed to more deleterious types of DNA damage [52]. For instance, protein–DNA complexes can be repaired via transcription-coupled repair, which has been shown to be enhanced for protein–DNA crosslinking compared to other types of damage [53]. In addition, Dsup has been shown to be constitutively expressed even in the absence of damage, thus it is bound to chromatin and DNA prior to any potential DNA damage.

There are several problems with this mechanism. First, it is unclear how this type of model would protect the DNA from reactive oxygen species. This is because reactive oxygen species and ionizing radiation, by contrast to ultraviolet radiation, are not likely to lead to protein–DNA crosslinks. As a result, this model would predict that Dsup would be more protective against ultraviolet radiation compared to ionizing radiation, which does not appear to be the case. Second, most protein–DNA crosslinks involve cysteines and tyrosine residues. As previously mentioned, Dsup has no cysteines and one tyrosine residue. Thus, any such crosslinks would need to occur via other side chains such as lysine and histidine, which are less reactive in comparison.

### 3.5. Molecular Scaffolding for DNA Repair

The second proposed indirect mechanism of Dsup protection involves Dsup acting as a molecular scaffold for enhanced recruitment of DNA repair proteins. According to this model (Figure 3F), Dsup would preferentially bind to sites of DNA damage or even scan along the DNA via transient, multivalent interactions. When it encounters a site of damage, Dsup would bind it with high affinity. While engaged with the damaged DNA, the unstructured Dsup protein could more efficiently recruit and organize DNA repair proteins.

Support for this model comes from two simple observations. First, it has been shown that Dsup binds some forms of DNA damage, such as abasic sites and 8-oxoguanines, with high affinity [39]. This tight binding would be advantageous to efficiently recruit DNA repair factors to sites of DNA damage. Second, the intrinsically disordered nature of Dsup is analogous to other key molecular scaffolds such as histone tails and the mediator complex in transcription that recruit other protein factors [38,39,40,41].

There are currently two key problems with this model. First, to our knowledge, there have been no studies showing that Dsup interacts with DNA repair proteins. Even if it does interact with DNA repair proteins, it is difficult to reconcile this model with the fact that Dsup confers protection to DNA damage in a wide range of eukaryotes. Dsup would not only have to interact with DNA repair factors in tardigrades, but it would also have to interact with these protein’s orthologs in yeast, fruit flies, mice, humans, rice, and tobacco, which seems highly unlikely given the diversity of these species. Second, Dsup has been shown to bind damaged DNA with similar high affinity as it binds undamaged DNA [39]. This would not allow for the high specificity for damaged DNA required by this model. However, this final point should be taken with some caution as damaged and non-damaged DNA substrates used in these binding experiments were produced by annealing two oligodeoxynucleotides. Thus, both the damaged and non-damaged DNA substrates resemble double-stranded DNA breaks with blunt ends, which Dsup might regard as damaged substrates, which it would bind with high affinity.

In summary, there have been a variety of mechanisms proposed for the protection of genomic DNA by Dsup. These models all seem to have some supporting evidence; however, each model has key gaps in our knowledge or problems that remain unresolved. As a result, we suggest that the most likely scenario is that the protective properties of Dsup arise from a combination of several of these models. Moreover, the effects of diverse types of damaging agents may be mitigated by different combinations of strategies. More research is required to untangle the mechanism of action of Dsup.

## 4. Applications of Dsup

The demonstration that Dsup protects DNA from radiation-induced damage when heterologously expressed in human cells has led researchers to consider novel potential biomedical applications [23,27,30,31,34,54]. For example, melanomas generally result from the exposure of skin to ultraviolet radiation [55,56]. Similarly, ischemia and strokes are often associated with elevated levels of oxidative stress within cells [57,58,59]. Since Dsup can protect cells from the deleterious effects of radiation and oxidative stress [23,27,29,34], heterologous expression of Dsup should allow for the prevention, progression, and perhaps treatment of these conditions. Despite the potential for Dsup to improve human health, caution is warranted, as Dsup expression in primary neuronal cells has been shown to promote DNA damage [26]. This demonstrates the need for more detailed work to assess and to determine the mechanism of such toxicity. While long-term, heterologous expression of Dsup in humans is likely far-fetched, the development of drugs that mimic the mechanism by which Dsup protects DNA could one day be clinically useful.

Another potential application of the heterologous expression of Dsup or the use of Dsup-mimicking drugs is in the radiation treatment of cancers. Dsup or Dsup-inspired drugs could be used to protect the healthy tissue surrounding a tumor. This should provide clinicians with the opportunity to expose cancers to high doses of radiation without excessive damage to the surrounding tissues. Recently, researchers showed that lipid nanoparticles can deliver mRNA encoding Dsup to tissues in mouse models. Moreover, this mRNA-based delivery approach confers radioprotection to healthy tissues in mice with orthotopic oral cancer undergoing radiation therapy [60].

In addition to human health, Dsup can aid in several economic sectors that directly impact human well-being. For example, Dsup can be used in agriculture to produce transgenic crops that are more resistant to radiation. As mentioned above, Dsup has already been shown to protect rice and tobacco plants from radiation induced damage [30,31]. As the global population grows and the level of crop damage continues to rise, radiotolerant crops may increasingly become important to feed the world.

Finally, as humans continue to explore our solar system, it will become necessary to protect people and their food sources from cosmic and solar radiation, because conditions in space, on the Moon, and on Mars—our most probable destinations—are not conducive for plant and animal life. Again, the heterologous expression of Dsup or the use of Dsup-mimicking drugs would be valuable in opening these future niches without the need for costly radiation shielding [61,62,63,64,65].

## 5. Conclusions

In this review, we summarized our current understanding of the cellular, functional, structural and biochemical properties of the tardigrade-specific Dsup protein. We described how the heterologous expression of Dsup protects a variety of diverse systems from diverse DNA-damaging stressors, highlighting the versatility and complexity of Dsup function. We summarized several key findings about the structural and biochemical properties of Dsup. We then described several models that could account for the DNA-protective properties of Dsup, and we discussed the supportive evidence and challenges associated with each model. Lastly, we examined several potential applications of Dsup or Dsup-inspired drugs.

In addition to Dsup, tardigrades likely possess other molecular adaptations and other proteins that contribute to their tolerance of extreme environmental stress [48,49]. One recent example is Tardigrade DNA Damage Response 1 (TDR1), another protein implicated in radiotolerance [66]. Like Dsup, TDR1 binds DNA and possesses an unusual primary sequence lacking cysteines and containing very few aromatic residues. The genes encoding for both TDR1 and the Dsup ortholog were identified in the *Hypsibius exemplaris* genome, a tardigrade subspecies known to be extremely tolerance to ionizing radiation. However, in contrast to Dsup, TDR1 is not constitutively expressed; instead, its expression is upregulated in response to radiation exposure [66]. These differences suggest that TDR1 may function through a distinct mechanism, potentially acting after DNA damage has already occurred. Although much less is known about TDR1 compared to Dsup, its discovery highlights the growing number of tardigrade-specific proteins that may contribute to extreme stress tolerance.

Since the discovery of Dsup ten years ago, progress in this field has been slow, and large gaps in our understanding of the mechanism of this protein remain. Fortunately, through several key findings—arguably the most notable being that heterologous expression protects a variety of animal and plant systems—interest in this field has been increasing rapidly. Now multiple groups of biochemists, biophysicists, and structural biologists are turning their attention to Dsup. We are optimistic that in the coming years, many of our knowledge gaps regarding Dsup will be filled. Hopefully, this will lead to Dsup-inspired strategies and drugs that will make some of the potential applications of Dsup realities.

## Data Availability

No new data were created or analyzed in this study. Data sharing is not applicable to this article.

## Abbreviations

The following abbreviations are used in this manuscript:

ROS	reactive oxygen species
Dsup	damage suppressor
HMGN	high motility group nucleosome-binding domain
SAXS	small-angle X-ray scattering
UV	ultraviolet
